# Nasopharyngeal Bacterial Interactions in Children

**DOI:** 10.3201/eid2002.121724

**Published:** 2014-02

**Authors:** Motoi Suzuki, Bhim Gopal Dhoubhadel, Lay Myint Yoshida, Koya Ariyoshi

**Affiliations:** Institute of Tropical Medicine, Nagasaki University, Japan

**Keywords:** bacterial colonization, acute otitis media, Haemophilus influenzae, Streptococcus pneumoniae, bacteria, respiratory infections, children

**To the Editor:** Xu and colleagues (*1*) examined the nasopharyngeal bacterial colonization rates in children with acute otitis media (AOM) and in healthy children. They found that *Haemophilus influenzae* colonization was competitively associated with *Streptococcus pneumoniae* and *Morexella catarrhalis* colonization in children with AOM but was not associated with *S. pneumoniae* and *M. catarrhalis* colonization in healthy children. We have a serious concern regarding their analysis.

The authors calculated odds ratios (ORs) by considering a bacterial colonization as an outcome variable and another bacterial colonization as an exposure variable. They considered an OR >1 as the presence of synergistic associations between bacteria (i.e., co-colonization is more likely to occur than it would by chance) and OR <1 as the presence of competitive associations (i.e., co-colonization is less likely to occur than it would by chance). This inference may be justified in a population-based cross-sectional study. If 2 bacterial colonizations occur independently in a stationary population, the prevalence of co-colonization will be the product (multiplication) of each prevalence, and the OR between 2 bacterial colonizations in the population (OR_pop_) will be 1 (Technical Appendix).

However, the authors enrolled their case-patients according to clinical signs (i.e., AOM or healthy). Let us assume that case-patients are enrolled from a population of children during a time period of *t*. Let r_c_ be the risk for enrollment (that is, of developing the disease) among colonized children and r_n_ be the risk for enrollment among noncolonized children. The OR among the enrolled case-patients (OR_case-patient_) becomes r_n_/r_c_, which is the reciprocal of the risk ratio (RR) of developing the disease (RR = r_c_/r_n_) (online Technical Appendix). Usually, RR is >1 for diseased children and <1 for healthy children. Therefore, even in this independent colonization scenario, OR_case-patient_ becomes <1 (“pseudo-competitive associations”) in diseased children, and OR_case-patient_ becomes >1 (“pseudo-synergistic associations”) in healthy children. This is probably what the authors have observed in the study.

We cannot infer an association of multiple bacterial colonizations in a population despite an observed association in the diseased (or healthy) children, and this association is widely misunderstood (*2*–*4*). The authors’ discussion regarding a potential emergence of *H. influenzae*, associated with AOM, after the introduction of pneumococcal conjugate vaccine is thus unjustifiable.

Technical Appendix Number of children by bacterial colonization status in a stationary population and case-patients enrolled during a specified period.
