# Supplementary material for: Nasopharyngeal Bacterial Interactions in Children
Source: Emerg Infect Dis. 2014 Feb;20(2):323–4. doi: 10.3201/eid2002.121724 (PMC3901485; doi:10.3201/eid2002.121724)
Supplement: Technical Appendix — Number of children by bacterial colonization status in a stationary population and case-patients enrolled during a specified period. [file 12-1724-Techapp-s1.pdf]

DOI: http//dx.doi.org/10.3201/eid2002.121774

Nasopharyngeal Bacterial Interactions in Children

Technical Appendix

Figure. Number of children by bacterial colonization status in a stationary population (A) and cases enrolled during a specified time period (B).

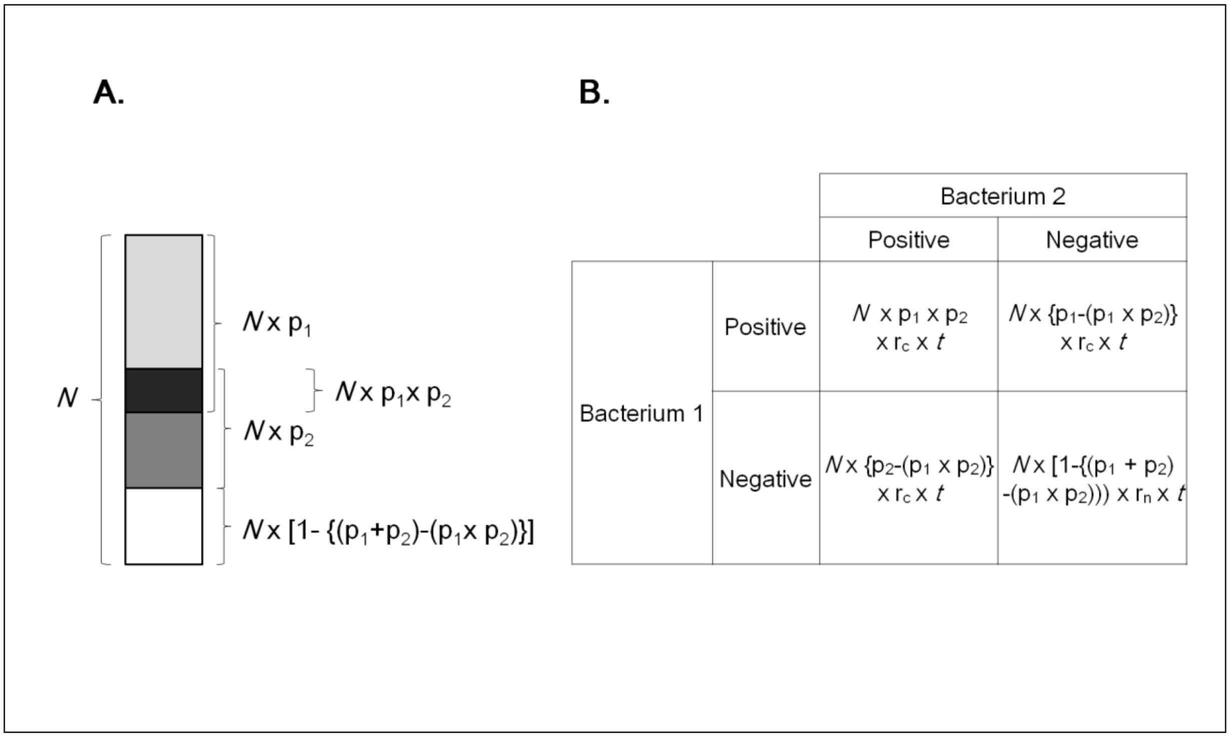

N: the population size

p_1: the prevalence of bacterium 1 colonization

p_2: the prevalence of bacterium 2 colonization

r_c: the risk of enrollment among colonization-positive children

r_n: the risk of enrollment among colonization-negative children

t: a study period

Suppose the colonization of bacterium 1 and of bacterium 2 occur independently in the population.

The odds ratio between bacterium 1 and bacterium 2 in the population (OR_{pop}) will be

$$OR_{pop} = \frac{N \times p_1 \times p_2 \times N \times [1 - \{(p_1 + p_2) - (p_1 \times p_2)\}]}{N \times \{p_1 - (p_1 \times p_2)\} \times N \times \{p_2 - (p_1 \times p_2)\}}$$

$$= \frac{p_1 \times p_2 - (p_1 \times p_2) \times (p_1 + p_2) - (p_1 \times p_2)^2}{p_1 \times p_2 - (p_1 \times p_2) \times (p_1 + p_2) - (p_1 \times p_2)^2}$$

$$= 1$$

The OR between bacterium 1 and bacterium 2 in the enrolled cases will be

$$OR_{case} = OR_{pop} \times \frac{r_c \times t \times r_n \times t}{r_c \times t \times r_c \times t}$$

$$= OR_{pop} \times \frac{r_n}{r_c}$$

$$= \frac{r_n}{r_c}$$

which is the reciprocal of risk ratio for enrollment (= developing the disease; r_c/r_n).
